# Dimensionality of the Fonseca Anamnestic Index and validation of its short-form derivative

**DOI:** 10.2340/aos.v84.42960

**Published:** 2025-03-18

**Authors:** Adrian Ujin Yap, Indrayadi Gunardi, Darren Zong Ru Lee, Carolina Marpaung

**Affiliations:** a Division of Dentistry, Ng Teng Fong General Hospital, Singapore, Singapore; b Faculty of Dentistry, National University Health System, Singapore, Singapore; c National Dental Research Institute, Singapore, Singapore; d National Dental Center, Singapore, Singapore; e Duke-NUS Medical School, Singapore Health Services, Singapore, Singapore; f Department of Prosthodontics, Faculty of Dentistry, Universitas Trisakti, Indonesia; g Department of Oral Medicine, Faculty of Dentistry, Universitas Trisakti, Jakarta, Indonesia; h School of Health and Social Sciences, Nanyang Polytechnic, Singapore, Singapore

**Keywords:** Temporomandibular disorders, general population, questionnaires, validity, methods

## Abstract

**Objectives:**

Recently, the Short-Form Fonseca Anamnestic Index (SFAI) was shown to have high diagnostic accuracy when compared to the Diagnostic Criteria for Temporomandibular Disorders (DC/TMD) in patient samples. This study investigated the dimensionality of the parent instrument (Fonseca Anamnestic Index [FAI]) and validated its main component using Rasch analysis in non-patient populations.

**Methods:**

FAI data from a total of 901 participants from Singapore and Indonesia with a mean age 19.30 ± 1.48 years (65.0% women) were examined. Of these, 53.8% were FAI positive and 46.2% were FAI negative. Principal Component Analysis (PCA) was performed to extract the main component of the FAI using an eigenvalue > 1.0 and direct oblimin rotation with item loading of > 0.40. Rasch analysis was subsequently carried out on the items of the main component.

**Results:**

The FAI was found to be multidimensional with the main component involving items F1, F2, F3, F6, and F7 which were the items of the SFAI. The SFAI had moderate internal consistency (Cronbach alpha = 0.63) and fitted the Rasch model with person and item infit/outfit mean square (MnSq) values of 0.98/0.96 and 1.00/0.96 logits respectively. The infit/outfit MnSq of the SFAI items ranged from 0.82 to 1.06 logits with Item F2 (side-movement difficulty) being the most difficult and item F3 (muscle pain) the easiest.

**Conclusions:**

The FAI is multidimensional with the main component comprising the five items of the SFAI that fitted the Rasch model. With its good Rasch validity, separation, and reliability, the SFAI is a promising tool for TMD screening.

## Introduction

Temporomandibular disorders (TMDs) are a diverse group of neuromuscular and musculoskeletal conditions affecting the temporomandibular joints (TMJs), muscles of masticatory, and adjoining structures. In addition to TMJ and masticatory muscle pain, the other signs/symptoms of TMDs include headaches, neck pain, TMJ noises, jaw functional limitations, as well as abnormal jaw movements [1, 2]. The complex etiology of TMDs involves a myriad of biopsychosocial risk factors including sex hormones, trauma, parafunctions, stress and emotions [1, 2]. Women, particularly those aged between 20 and 40 years, appear to be at greater risk of TMDs [1–3]. While the prevalence of TMDs established by formalized diagnostic criteria ranged from 6.0 to 15.8%, the occurrence of TMD signs/symptoms in the general population was substantially greater (up to 75.0%) based on clinical evaluations/self-reported questionnaires [4, 5]. The high variability in TMD prevalence observed may be explained by the different instruments/protocols employed for identifying and diagnosing TMDs.

Formalized diagnostic criteria, like the Research Diagnostic and Diagnostic Criteria for TMDs (RDC/TMD and DC/TMD), generally involve comprehensive symptom history taking, protocolized physical examination, and defined algorithms for rendering specific TMD diagnosis [6, 7]. Though reliable and valid, the use of RDC/TMD and DC/TMD is not pragmatic for clinical triage and epidemiological studies involving large samples due to their complicated and time-consuming procedures. Instruments for screening the presence/absence of TMDs or ‘TMD screeners’ need to be cheap, easy, fast to dispense (ideally self-administered), reliable, and valid. Gonzalez et al. had reviewed the shortcomings of past TMD screeners and current ones include the TMD Pain Screener (TPS), Three Questions for TMDs (3Q/TMD), and Fonseca Anamnestic Index (FAI) [8–10]. The 6-item TPS is a component of the DC/TMD, but is specifically designed to identify painful TMDs. In contrast, the 3-item 3Q/TMD and the 10-item FAI are capable of detecting both pain-related and intra-articular conditions. However, the diagnostic accuracy of the 3Q/TMD might be constrained due to its limited number of questions [8, 9]. Among the three screening instruments, the FAI is the most widely used and has been translated into numerous languages, including Chinese, Malay, Turkish, Arabic, Spanish, and Polish [11–16].

The psychometric properties of the FAI are well-reported and it was shown to yield consistent outcomes with other TMD screeners such as the American Association of Orofacial Pain Questionnaire as well as formalized TMD diagnostic criteria [11–20]. More recently, the Short-Form Fonseca Anamnestic Index (SFAI), comprising of five TMD-specific items, demonstrated high diagnostic accuracy when related to both the RDC/TMD and DC/TMD [20, 21], raising concerns over the possible over-estimation of TMD prevalence by the FAI due to its multidimensionality (measurement of more than one dimension of a construct) and inclusion of non-TMD-specific symptoms and risk factors, specifically headache, neck pain, parafunctions, malocclusion, and emotional tension [22].

The objectives of this study were to explore the dimensionality of the FAI and to validate its main component using Rasch analysis. The latter is a modern statistical technique founded on the item-response theory that is commonly employed in medicine for instrument development and refinement [23]. The Rasch model stipulates that item responses are the result of linear probabilistic interactions between the person’s ‘ability’ and the item’s ‘difficulty’ [24]. It transforms ordinal data into interval-scaled ones that are conveyed in ‘log odds/logits’ and hierarchically arranged. In addition, the Rasch model indicates fit statistics specifying how well discrete items and individual respondents describe/match the group [25]. The null hypotheses were: (1) The FAI is not multidimensional, and (2) items of its main component do not fit the Rasch model and are not valid in terms of person ability and item difficulty.

## Materials and methods

Data for this study were gathered from health surveys approved by the relevant institutional review boards (reference numbers: SHS2018005 and 377-S1/KEPK/FKG/8-2020). The participants were recruited from young adults attending a polytechnic in Singapore and University in Indonesia. A minimum sample of 371 participants was established using a sample size calculator (https://www.calculator.net) founded on a 95% confidence level, 5% confidence interval, a combined student population of 34,700, and 42% mild-to-severe TMD based on the FAI [26]. Individuals must be aged 18–44 years and proficient in English or Bahasa Indonesia (BI) to qualify for the study. Those with a history of recent (prior 2 weeks) orofacial trauma/surgical procedures, debilitating psychological, and systemic conditions were excluded. Participation in the study was voluntary with no incentives offered. Informed consent was obtained before completing questionnaires that included demographic information and the English or Indonesian language versions of the FAI. The Indonesian FAI (FAI-I) was developed following the International Network for Orofacial Pain and Related Disorders Methodology (INfORM) protocol and has good reliability and validity [27].

The FAI comprised of four pain-related questions (TMJ, masticatory muscle, neck pain, and headaches), three function-related questions (TMJ sounds, opening, and side-movement difficulty), and three questions on TMD risk factors (parafunctional habits, malocclusion, and emotional tension). Participants rated the FAI items on a 3-point response scale with ‘no’, ‘sometimes’, and ‘yes’ being awarded 0, 5, and 10 points, respectively. Total sum scores are calculated and TMD severity is graded as follows: ‘no’ (0–15 points), ‘mild’ (20–40 points), ‘moderate’ (45–65 points), or ‘severe’ (70–100 points) [10, 17]. For the SFAI, sum scores are only totaled for items F1, F2, F3, F6, and F7. The absence and presence of TMDs are indicated by scores of ≤ 15 points and ≥ 20 points, respectively [20, 21].

Statistical and Rasch analyses were conducted using the SPSS statistics software version 24.0 (IBM Corporation, Armonk, New York, USA) and Winsteps Version 4.3.4 (Linacre, Beaverton, Oregon, USA) [28]. Principal Component Analysis (PCA) was utilized to establish the dimensionality of the FAI. To confirm the applicability of PCA, sampling adequacy was assessed through the Kaiser-Meyer-Olkin (KMO) test, and the significance of item correlations was examined using Bartlett’s test of sphericity (BTS). While KMO values ≥ 0.60 are considered satisfactory, BTS *p*-values of < 0.05 indicate adequate item correlations and suitability for factor extraction. An eigenvalue > 1.0 and direct oblimin rotation with a threshold item loading of > 0.40 were applied for extracting the components of the FAI [29]. The main component that explained most of variance was isolated and its internal consistency (Cronbach alpha [α]) was calculated and categorized as follows: very low (α ≤ 0.30); low (0.30 < α ≤ 0.60); moderate (0.60 < α ≤ 0.75); high (0.75 < α ≤ 0.90); and very high (α > 0.90) [30].

To validate the main component of the FAI, Rasch analysis was performed, focusing on person/item fit (the alignment of individual responses and test items with the expected measurement model), separation (the ability to distinguish between different levels of the trait being measured), reliability statistics (the consistency of the measurement across respondents and items), and point-measure correlations (the relationship between individual item scores and the overall trait being measured). Fit statistics, including infit (sensitivity to unexpected responses to items close to a respondent’s ability level) and outfit (sensitivity to unexpected responses to items far from a respondent’s ability level) values, were reported as mean-square (MnSq) statistics. Acceptable MnSq ranges for polytomous models (response options ≥ 3) were 0.6–1.4 logits, with standardized fit statistics (Zstd) within ± 1.9 [29]. Items with MnSq values outside this range (< 0.6 or > 1.4 logits) were excluded. Item difficulty was assessed using θ values, and participants’ ability was analyzed using the Wright person-item map [24, 25]. Probability curves for the three response categories were plotted, and differential item functioning (DIF) was checked by country (Singapore/Indonesia) and gender (female/male) [31, 32].

## Results

Out of 1219 eligible young adults screened, 318 declined study involvement ensuing in a response rate of 73.9%. The mean age of the 901 participants, comprising 400 Singaporeans and 501 Indonesians, was 19.30 ± 1.48 years and 65.0% were women. Of these, 46.2% (*n* = 416) were FAI-negative and 53.8% (*n* = 485) were FAI-positive. Mild, moderate, and severe TMD was present in 43.2%, 9.9%, and 0.8% of the participants accordingly (Supplementary Table 1). Based on the SFAI, TMD was present in 10.8% of the cohort and absent in 89.2%.

Items of the FAI demonstrated adequate sampling and appropriate correlations for PCA with a KMO score 0.77 and a significant BTS (*p* < 0.001). Table 1 displays the outcome of PCA. The FAI was found to be multidimensional with three components explaining 50.9% of the scale variance. Items F1, F2, F3, F6, and F7, which were the items of the SFAI, formed the main component and accounted for 27.1% of the difference. While the second component consisted of items F4, F5, and F10, the third component involved items F8 and F9. The internal consistency of the main component of SFAI was moderate with a Cronbach alpha (α) of 0.63 (Table 2).

**Table 1:** Dimensionality of the FAI (*n* = 901).

Items^a^		Component		
		1	2	3
F1	Do you have difficulty opening your mouth wide?	**0.83**		
F2	Do you have difficulty moving your jaw to the sides?	**0.75**		
F7	Have you ever noticed any noise in your TMJ while chewing or opening your mouth?	**0.60**		
F6	Do you have ear aches or pain in that area?	**0.41**		
F3	Do you feel fatigue or muscle pain when you chew?	**0.41**		
F4	Do you have frequent headaches?		**0.83**	
F5	Do you have neck pain or a stiff neck?		**0.77**	
F10	Do you consider yourself a tense (nervous) person?		**0.50**	
F8	Do you have any habits such as clenching or grinding your teeth?			**0.76**
F9	Do you feel that your teeth do not come together well?			**0.73**
Eigenvalues		2.71	1.37	1.01
Scale variance (%)		27.14	13.74	10.05
Total scale variance (%)		50.93		

Values in bold highlight factor loadings that correspond to the specific component most. Component loadings < 0.40 were omitted. FAI: Fonseca Anamnestic Index; TMJ: Temporomandibular joints.

**Table 2:** Summary of factor loadings of the items in the main component of the FAI (SFAI).

Items	Component 1
F1. Do you have difficulty opening your mouth wide?	0.72
F2. Do you have difficulty moving your jaw to the sides?	0.67
F7. Have you ever noticed any noise in your TMJ while chewing or opening your mouth?	0.66
F6. Do you have ear aches or pain in that area (temporomandibular joint)?	0.60
F3. Do you feel fatigue or muscle pain when you chew?	0.59
**Eigenvalue**	2.10
**% of variance explained**	41.94
**Cronbach alpha (KR-20)**	0.63

Values ≥ 0.40 were considered for relevant factor loadings after oblique rotation. FAI: Fonseca Anamnestic Index; SFAI: Short-Form Fonseca Anamnestic Index; TMJ: Temporomandibular joints.

Table 3 shows the outcomes of Rasch analysis including fit index for persons/items and the degree of difficulty for the SFAI items. Mean person (participant) ability was –1.48 ± 0.94 logits with infit and outfit MnSq values of 0.98 and 0.96 logits. Mean item difficulty was 0.00 ± 0.75 logits with infit and outfit MnSq values of 1.00 and 0.96 logits. While Rasch person separation was low (0.00), Rasch item separation was high (8.05). Similarly, Rasch person reliability was also low (0.00), but Rasch item reliability was excellent (0.98). All five items of the SFAI fitted the Rasch model. Infit MnSq values ranged from 0.87 to 1.06 logits, whereas outfit values varied from 0.82 to 1.03 logits. Infit and outfit Zstd values were within the span of ± 1.9. Point-measure correlations were all positive and fluctuated between 0.50 and 0.71. Item F3 (Do you feel fatigue or muscle pain when you chew?) was observed to be the easiest (**θ** = –0.80) and item F2 (Do you have difficulty moving your jaw to the sides?) was deemed the hardest (**θ** = 1.25). Table 4 indicates the differential item functioning for country and gender. Country bias was observed for item F3 and gender bias was noted for items F3 (muscle pain) and F6 (TMJ pain) (*p* < 0.05).

**Table 3:** Fit index for persons/items and degree of item difficulty (logits).

Item	Measure	Misfit order				
		Infit MnSq	Infit Zstd	Outfit MnSq	Outfit Zstd	Point-measure correlation
**Summary of persons** Mean ± SD	-1.48 ± 0.94	0.98	0.09	0.96	0.11	-
**Summary of items** Mean ± SD	0.00 ± 0.75	1.00	0.12	0.96	-0.20	-
**Item statistics**	**(θ)**					
F1	0.13	0.87	-1.92	0.93	-0.84	0.62
F2	1.25	1.00	0.00	0.82	-1.20	0.50
F3	-0.80	1.04	0.64	1.01	0.21	0.68
F6	0.18	1.06	0.87	1.03	0.35	0.58
F7	-0.76	1.06	1.02	1.03	0.49	0.71

MnSq: mean-square; Zstd: Z-standardized; θ: theta (item’s difficulty index).

**Table 4:** Differential item functioning for country and gender (*n* = 901).

Item	Country		Gender	
	X^2^	Prob	X^2^	Prob
F1	0.880	0.348	2.082	0.149
F2	0.289	0.590	1.842	0.174
F3	4.104	0.042*	20.101	0.000*
F6	1.395	0.237	3.893	0.048*
F7	3.627	0.056	0.768	0.380

* Results of Chi-square test (*p* < 0.05).

**Figure 1:**
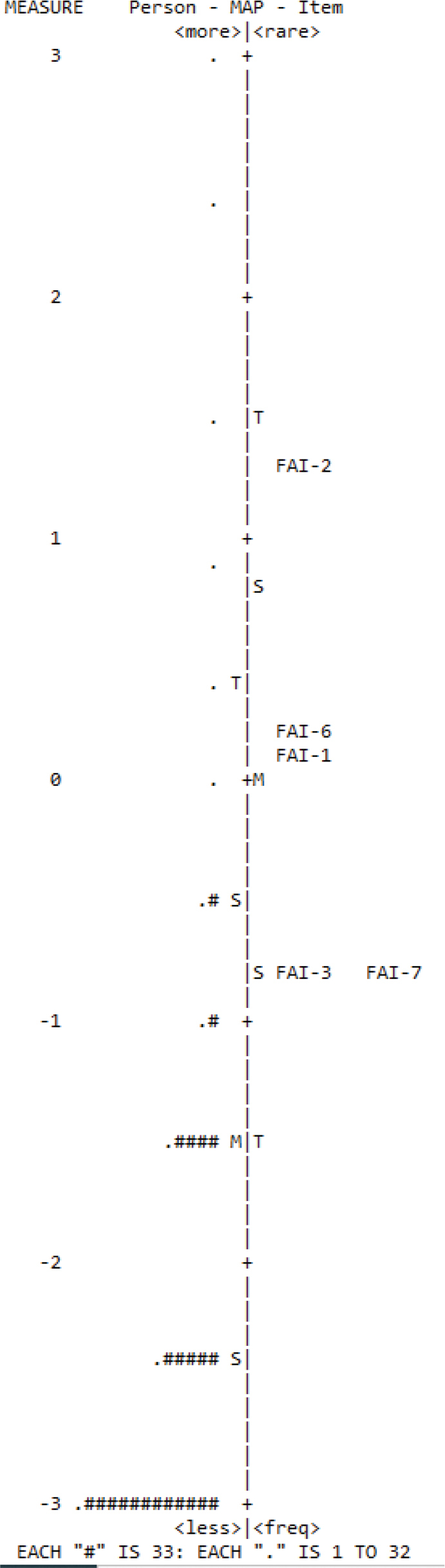
Wright person-item map for the items of the main component of the FAI (SFAI). Right side indicates item difficulty while left side indicates person ability. M: mean; S: 1 SD; T: 2 SD; FAI: Fonseca Anamnestic Index; SFAI: Short-Form Fonseca Anamnestic Index.

**Figure 2:**
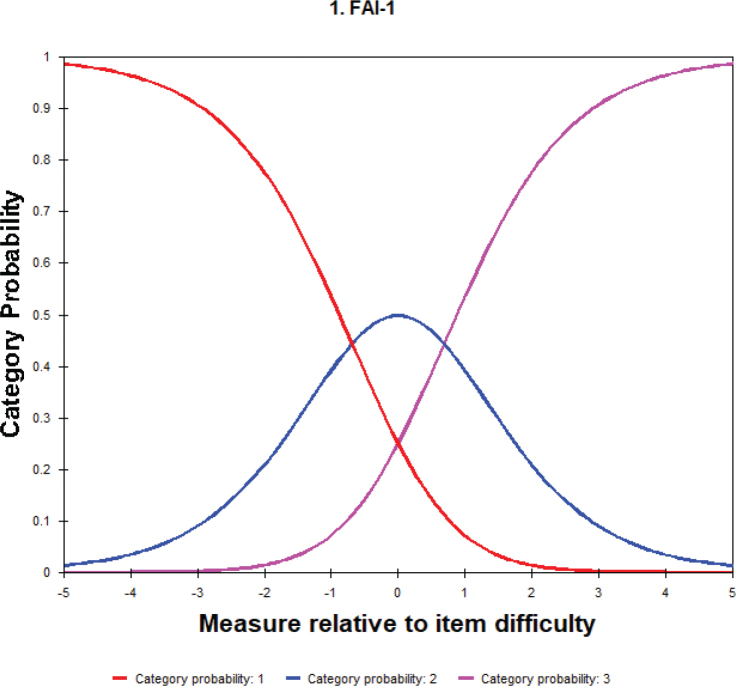
Probability curve to show the operation of the three response categories (category 1 = no, category 2 = sometimes, and category 3 = yes).

The Wright person-item map is presented in Figure 1 with person and item measures indicated on the left and right sides, respectively. Person measure is ordered based on ability (greater TMD severity on top) whilst item measure is ordered according to difficulty (more challenging on top). Person measure extended from –3.75 to 3.75 logits whereas item measure went from –0.80 to 1.25 logits. The spread of person ability surpassed that of item difficulty and was partial toward the lower end of TMD severity. The mean (M) item difficulty was higher than the mean (M) person ability suggesting that the items generally have a lower prospect of being endorsed aptly by the study sample. Figure 2 displays the probability curves for the three response categories. All response categories had distinct peaks with Rasch-Andrich thresholds ranging from –0.69 to 0.69 specifying that they were the most probable response category and applicable for defining the measured variables.

## Discussion

The dimensionality of the FAI was ascertained and its main component was validated using Rasch analysis. As the FAI was multidimensional and the items of its main component (SFAI) conformed to the Rasch model in terms of fit, separation, and reliability statistics, both null hypotheses were rejected. This study was the first to jointly appraise two language versions of the FAI allowing for more precise estimates and generalization of results for Southeast Asian non-clinical community samples. The Indonesian FAI had suitable test-retest reliability (intra-class correlation coefficient = 0.72) and validity when related to the Oral Health Impact Profile-14 (correlation coefficient = 0.47) [33]. Young adults were selected for the study as they represent the peak incidence age for TMDs and constitute the majority of TMD patients [1, 2, 34]. Among the young adults examined, 53.8% were FAI-positive with the majority (46.2%) having mild TMD. The finding corroborated those of similar Asian studies reporting FAI-based TMD prevalence ranging from 46.8 to 53.3% [35, 36]. These prevalence rates were considerably greater than those established using formalized diagnostic criteria. Based on the SFAI, TMD was present in 10.8% of the participants which was consistent with the prevalence range (6.0–15.8%) determined with the RDC/TMD [2]. The aforementioned, reinforced concerns over the over-estimation of TMD prevalence with the FAI.

Multidimensionality of the FAI was confirmed with PCA, though a bifactor structure had also been reported [15]. The main component of the FAI consisted of the five items of the SFAI and verified the work of Rodrigues-Bigaton et al. who concluded that the main dimension of the FAI involved items F1, F2, F3, F6, and F7 [22]. These SFAI items had moderate internal consistency (α = 0.63) and were subsequently subjected to Rasch measurements. The second component encompassed items F4 (headache), F5 (neck pain), and F10 (emotional tension). Head and neck pain often co-exist arising from trigemino-cervical relationships and are associated with psychosocial factors including emotional distress [37, 38]. The last component entailed items F8 (parafunctional habits) and F9 (malocclusion) that are TMD risk factors. Sánchez-Torrelo et al. also identified three components in the FAI, though the distribution of items differed slightly [12]. While systematic reviews indicated that parafunctions such as awake/sleep bruxism could be related to TMDs, no ‘clinically relevant’ associations were established between dental malocclusion and TMDs in population-based and clinical studies [39–41].

Classical test methods have been applied to practically all studies appraising the psychometric properties of the FAI [11–17]. However, the classical test theory has some conceptual restrictions. These include the supposition that variance in responses is due to person ability, reliability remains constant for different person ability, and all items contribute equally to the total score [42, 43]. In contrast to the classical test approach, Rasch analysis supports the examination of fit, separation, and reliability statistics, and hierarchical structures, and provides distribution as well as probability graphics [44]. MnSq fit statistics specify the magnitude of randomness with values < 0.6 and > 1.4 logits indicating unfavorable data predictability and unpredictability, respectively. Both infit (inlier-sensitive) and outfit (outlier-sensitive) MnSq for person ability, item difficulty, and all five items of the SFAI were within the ideal range of 0.6–1.4 logits and were productive for measurement. Furthermore, infit/outfit Zstd values were within the range of ±1.9 indicating that the data had reasonable predictability [31]. The low person separation (< 2) implies that the SFAI is not sufficiently sensitive to distinguish individuals with mild and severe TMD. This was consistent with the purpose of the SFAI which was to detect the presence or absence of TMDs. The high item separation (> 3) suggests that the person sample was large enough to confirm the construct validity (item difficulty hierarchy) of the SFAI. The low person reliability observed can be attributed to the very low occurrence of moderate-to-severe TMD (7.7%) among the young adults examined. The excellent reliability of SFAI indicates that it had a large item difficulty range and a reproducible item difficulty hierarchy. Point-measure correlations were all positive denoting that all SFAI item-level scoring concurred with the latent variables.

Item F3 (muscle pain) and item F2 (side-movement difficulty) were considered the least and most difficult questions by the participants. The findings are plausible considering that the mean glide distance during mastication is under 1.5 mm and participants may have problems recognizing limited lateral jaw movements [45]. As regards differential item functioning, country bias was present for item F3 and gender bias was present for items F3 and F6 (TMJ pain). The country bias for item F3 can be ascribed to the higher frequency of self-reported muscle fatigue/pain in Indonesian young adults (39.1%) compared to their Singaporean (19.6%) counterparts. Similarly, the gender bias could be attributed to the higher risk of TMDs amongst women and their susceptibility to chronic pain conditions [1, 46]. While muscle pain was reported by 35.0% of women and 22.2% of men, TMJ pain was reported by 19.1% of women and 12.7% of men. The greater pain sensitivity in women had been attributed to sociocultural, psychological, and experiential gender differences among other factors [46].

As shown in the Wright person-item map, the spread of person ability was greater than that of item difficulty and was partial to the lower end of TMD severity. This can again be ascribed to very low prevalence of moderate-to-severe TMD and the absence of TMD in the vast majority of subjects (89.2%) based on the SFAI. As the mean item difficulty was higher than mean person ability, items of the SFAI had a lower prospect of being endorsed appropriately by community samples without TMDs. This was in agreement with the greater positive predictive values (99.4–99.5%) than negative predictive values (41.7–83.3%) when the SFAI was referenced to the DC/TMD. The SFAI is thus more proficient at identifying the presence than the absence of TMDs [21].

This study, like all others, has its limitations. First, as a self-administered instrument, the FAI is susceptible to various biases, including those related to sampling techniques, social desirability, recall periods, participants’ understanding, and selective memory [47]. However, Rasch analysis helps mitigate some of these concerns by rigorously evaluating the psychometric properties of questionnaires, reducing measurement error, and ensuring that the data align with the underlying model. Second, only the English and Indonesian versions of the FAI were investigated. As dimensionality of the FAI may be dependent on instrument translation and the study population, future work should incorporate other language versions of the FAI and racial groups. Third, the study sample involved only young adults in the community. The study should be extended to older adults as well as the elderly to determine the reliability and validity of the SFAI in the general population. Lastly, it is important to note that the SFAI serves only as a TMD screener. The use of formalized diagnostic protocols and criteria, as well as adjunctive diagnostic imaging, are necessary for rendering definitive TMD diagnoses. Notwithstanding, the SFAI, with its simplicity, low cost, good validity, and reliability, is useful for identifying the presence of TMDs in clinical practice. Given the high prevalence of TMDs in the general population, clinicians should screen all patients for TMDs and document any pre-existing signs or symptoms before initiating treatment, particularly in light of the litigious nature of modern society.

## Conclusion

Within the limitation of this study, the following conclusions were drawn:

1. The FAI was multidimensional with the main component consisting of items F1, F2, F3, F6, and F7 which were the items of the SFAI.
2. The SFAI fitted the Rasch model and all its items were productive for measurement and had reasonable predictability.
3. The SFAI had high item separation confirming its construct validity and excellent item reliability with a large item difficulty hierarchy.
4. Item F3 (muscle pain) and item F2 (side-movement difficulty) were deemed the least and most difficult.
5. The SFAI can be used to screen for TMDs in clinical practice.

## Supplementary Material

Dimensionality of the Fonseca Anamnestic Index and validation of its short-form derivative
