# Supplementary material for: Dimensionality of the Fonseca Anamnestic Index and validation of its short-form derivative
Source: Acta Odontol Scand. 2025 Mar 18;84:42960. doi: 10.2340/aos.v84.42960 (PMC11971946; doi:10.2340/aos.v84.42960)
Supplement: Dimensionality of the Fonseca Anamnestic Index and validation of its short-form derivative [file AOS-84-42960-s1.pdf]

Supplementary material has been published as submitted. It has not been copyedited or typeset by Acta Odontologica Scandinavica.

Supplementary Table 1. Demographics and distribution of the study participants.

**Demographics / TMD severity**	**Total sample**	**Singapore**	**Indonesia**
**n (%)**	901 (100)	400 (44.4%)	501 (55,6%)
**Age**			
Mean ± SD	19.30 ± 1.48	18.77 ± 1.54	19.73 ± 1.27
Median (IQR)	19.00 (2)	19.00 (1)	19.00 (2)
**Gender**			
Women n (%)	586 (65.0%)	209 (52.3%)	377 (75.2%)
Men n (%)	315 (35.0%)	191 (47.8%)	124 (24.8%)
**FAI-negative** n (%)	416 (46.2%)	212 (53.0%)	204 (40.7%)
**FAI-positive** n (%)	485 (53.8%)	188 (47.0%)	297 (59.3%)
Mild	389 (43.2%)	139 (34.8%)	250 (49.9%)
Moderate	89 (9.9%)	45 (11.3%)	44 (8.8%)
Severe	7 (0.8%)	4 (1.0%)	3 (0.6%)
